# Fluctuation of depressive symptoms in cognitively unimpaired participants and the risk of mild cognitive impairment 5 years later: Results of the Heinz Nixdorf Recall study

**DOI:** 10.3389/fnbeh.2022.988621

**Published:** 2022-10-25

**Authors:** Martha Jokisch, Sara Schramm, Christian Weimar, Susanne Moebus, Janine Gronewold, Nico Dragano, Karl-Heinz Jöckel

**Affiliations:** ^1^Department of Neurology, University Hospital Essen, University of Duisburg-Essen, Essen, Germany; ^2^Institute for Medical Informatics, Biometry and Epidemiology, University Hospital Essen, University of Duisburg-Essen, Essen, Germany; ^3^BDH-Klinik Elzach gGmbH, Elzach, Germany; ^4^Institute for Urban Public Health, University Hospital Essen, University of Duisburg-Essen, Essen, Germany; ^5^Centre for Health and Society, Medical Faculty, Institute of Medical Sociology, University of Düsseldorf, Düsseldorf, Germany

**Keywords:** depression, mild cognitive impairment, prevention, cognitive decline, epidemiology

## Abstract

**Background:**

Depression might be an independent risk factor for cognitive decline, a prodromal dementia symptom or a reaction to cognitive/functional impairment.

**Objective:**

To investigate the association between (1) depressive symptoms and (2) depressive symptom patterns over 13 years with incident mild cognitive impairment (MCI) 5 years later.

**Materials and methods:**

We included 724/823 cognitively unimpaired men/women who participated in the population-based Heinz Nixdorf Recall study (t1: 2005–2008, ø62.9 years; t2: 2010–2015, ø68.1 years). Depressive symptoms were assessed in the study center and during six postal follow-ups using the short form of the Center for Epidemiologic Studies Depression Scale (CES-D). Relative risks (RR; 95% confidence intervals) for MCI at t2 (men/women: 71/76) were estimated for CES-D at t1 (linear and dichotomized at ≥17, cut-off for clinically relevant depressive symptoms) and CES-D fluctuations over 13 years (stable low, large fluctuations, stable high/stable around cut-off) using log-linear regression models with Poisson working likelihood adjusted for age, sex, education, diabetes mellitus, coronary heart disease, and stroke.

**Results:**

Fully adjusted risk for MCI at t2 (per CES-D point increase at t1) was elevated for the total cohort (1.053, 1.031–1.076), men (1.046, 1.012–1.081), and women (1.059, 1.029–1.090). Applying the dichotomized CES-D, risk for MCI was substantially increased for the total cohort [2.22 (1.38–3.58)] and in women [2.59 (1.46–4.58)]. Large CES-D fluctuations and stable high/stable around cut-off were associated with increased RR for MCI in the total cohort and in women compared to stable low symptoms.

**Conclusion:**

Depressive symptoms predicted MCI in cognitively unimpaired participants of our population-based study. Adequate treatment of depression may therefore contribute to the maintenance of normal cognition and delay dementia onset.

## Introduction

Depression is one of the most common mental disorders worldwide with more than 264 million people affected (GBD 2017 Disease and Injury Incidence and Prevalence Collaborators, 2018). Growing evidence suggests that depression is a risk factor for dementia (Jorm, 2001; Ownby et al., 2006; Almeida et al., 2017). In 2018, the number of people affected by dementia was 50 million worldwide and will triple to 152 million by 2050 (Patterson, 2018). Pathological changes of Alzheimer’s disease (AD) precede the onset of cognitive decline by 20–30 years (Jansen et al., 2015; Jack et al., 2018). Thus, the identification of persons at risk, especially while cognitively normal, is of major importance to delay or even prevent cognitive decline. Assessing depressive symptoms therefore might help to identify individuals at high risk of progression to AD (Dafsari and Jessen, 2020). Relevant biological mechanisms linking depression to dementia include (1) higher level of cortisol due to hyperactivity of the hypothalamic-pituitary-adrenal (HPA) axis leading to hippocampal atrophy (Frodl and O’Keane, 2013), (2) inflammatory changes (Dowlati et al., 2010) that *inter alia* decrease brain-derived neurotropic factor (Heneka et al., 2015), (3) increased amyloid production and deposition of amyloid plaques (Gatchel et al., 2019) [one of the hallmark pathologies of AD (Jack and Holtzman, 2013)], and (4) cerebrovascular disease that may lead to structural damage in frontal-striatal or frontal-executive circuits (Rashidi-Ranjbar et al., 2020). Depression could not only be a risk factor for dementia, but is also an early symptom or prodromal state of dementia. It might occur as a reaction to cognitive and functional impairment, or might be associated with a underlying common risk factor like stroke or other cerebrovascular diseases (Barnes et al., 2012; Gao et al., 2013; Dafsari and Jessen, 2020). In every mentioned scenario, depression seems to be an important accelerating factor of clinical progression and conversion from a preclinical stage to mild cognitive impairment (MCI) and finally to dementia (Dafsari and Jessen, 2020). There is robust longitudinal evidence for the association between depression and incident dementia (AD or vascular dementia) showing an approximately twofold increased risk (Diniz et al., 2013; Cherbuin et al., 2015; Ford et al., 2018; Livingston et al., 2020). Regarding MCI, the majority of studies found depression to be a contributor to the conversion of cognitively healthy participants to MCI (Barnes et al., 2006; Geda et al., 2006; Steenland et al., 2012; Gao et al., 2013). There is less evidence regarding fluctuations of depressive symptoms over time and the development of MCI. One would assume that recurrent depressive episodes or stable high depressive symptoms result in greater cognitive decline (Geda et al., 2006; Barnes et al., 2012). Dotson et al. (2010) observed a proportional increase in risk for all-cause dementia and AD as a function of the number of depressive episodes. However, recurrence of depression did not increase the risk of incident MCI. A previous analysis showed no association between MCI and a positive history of lifetime depression without current depressive symptoms (Dlugaj et al., 2015). Whether different patterns of depressive symptoms over time (i.e., stable depressive symptoms) influence the probability of MCI remains to be elucidated.

The aim of the present study was (1) to examine the association between depressive symptoms and incident MCI 5 years later in cognitively unimpaired participants, and (2) to examine the association of depressive symptom patterns over 13 years (stable high, stable low, stable around cut-off, large fluctuations) and incident MCI in a prospective population-based cohort study.

## Materials and methods

### Study participants

**FIGURE 1:**
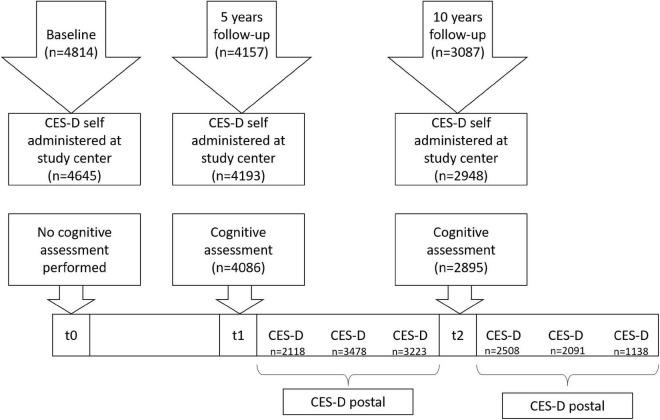
Timeline of the Heinz Nixdorf Recall study, Center for Epidemiologic Studies Depression Scale (CES-D), and cognitive assessments [for details see Engel et al. (2020)].

Participants of the prospective population-based Heinz Nixdorf Recall (Risk Factors, Evaluation of Coronary Calcification, and Lifestyle; HNR) study were randomly sampled from mandatory city registries in the Ruhr area in Germany and invited to participate in the study as previously reported (Schmermund et al., 2002; Stang et al., 2005). The major aim of the HNR study is to evaluate the predictive value of coronary artery calcification using electron-beam computed tomography for myocardial infarction and cardiac death in comparison to other cardiovascular risk factors (Schmermund et al., 2002; Stang et al., 2005). Briefly, 4,814 participants aged 45–75 years were enrolled in the baseline examination (t0) between 2000 and 2003. Participants were invited for follow-up examinations every 5 years (t1, *n* = 4157, 2005–2008, median follow-up time between t0 and t1: 5 years; t2, *n* = 3087, 2010–2015, median follow-up time between t0 and t2: 10 years). Data assessment included standardized interviews, clinical examinations, comprehensive laboratory tests, and self-administered questionnaires. A standardized cognitive performance assessment was introduced at t1 and was extended for t2 (see Section “Cognitive assessment”). In addition to the follow-up examinations in the study center, participants received yearly postal follow-up questionnaires. Depressive symptoms were assessed three times in the study center (t0, t1, and t2) and during six postal follow-ups using the short form of the Center for Epidemiologic Studies Depression Scale [CES-D (Radloff, 1977; Hautzinger and Bailer, 1993), see “Assessment of depressive symptoms”]. Figure 1 shows a timeline of the HNR study and time-points of CES-D assessments [for details see Engel et al. (2020)].

**FIGURE 2:**
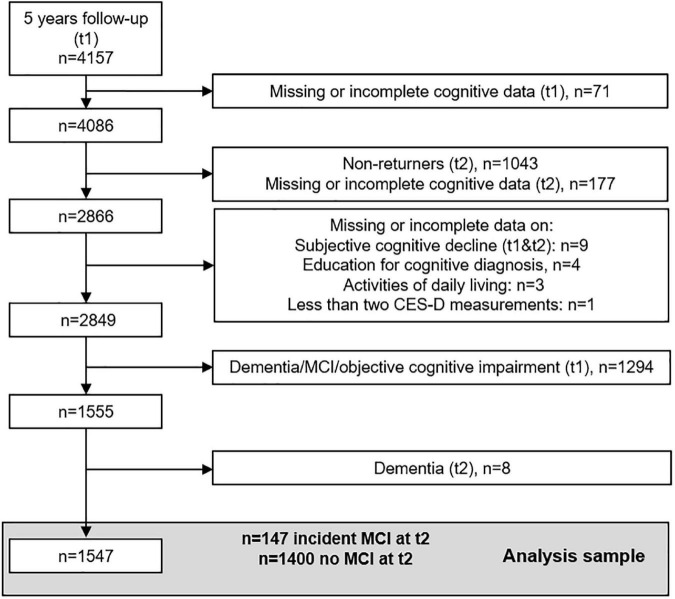
Sample flowchart for the present study. MCI, mild cognitive impairment; t1, first follow-up examination; t2, second follow-up examination.

Figure 2 shows a flow-chart of participants included in this analysis starting at t1 (*n* = 4,157), when cognitive assessment was implemented. Due to missing or incomplete cognitive data at t1, 71 participants were excluded. Of the remaining 4,086 participants, 1,043 did not return at t2 and 177 participants had missing or incomplete cognitive data at t2 resulting in 2,866 participants. Seventeen participants were excluded due to missing or incomplete data on subjective cognitive decline (SCD), education, activities of daily living or due to fewer than two CES-D measurements. As we were interested in incident MCI in previously (t1) cognitively healthy participants, we further excluded 947 participants with objective impairment not meeting the MCI criteria, and 341 participants with MCI and 6 demented participants at t1. Of the remaining 1,555 participants, 8 were demented at t2 (see Section “Cognitive diagnosis”). Thus, 1,547 participants (147 with incident MCI at t2 and 1,400 with no MCI at t2) were included in the analysis.

All participants provided written informed consent. The study was approved by the institutional review board of the University Duisburg-Essen and followed established guidelines of good epidemiological practice.

### Cognitive assessment

Briefly, the extended cognitive performance assessment at t2 consisted of eight subtests: (1) immediate and (2) delayed word list, (3) Labyrinth test, (4) verbal fluency “animals,” (5) clock-drawing test, (6, 7) Trail Making Test A and B, (8) Color-word test (card 1, card 2, difference card 3–card 2). For a detailed description of tests (1) to (5) see Wege et al. (2011) and for the extended cognitive assessment (6) to (8) see Tebrügge et al. (2018) and Müller-Gerards et al. (2019). Tests were grouped into the following four domains: (1) attention–Trail Making Test A, Color-word test card 1 and card 2; (2) executive function–Trail Making Test B, Labyrinth test, Color-word test interference performance, verbal fluency; (3) verbal memory–eight word list immediate and delayed recall; (4) visuoconstruction–clock-drawing test (Müller-Gerards et al., 2019). Cognitive impairment was defined as a performance of more than one SD below the age- and education adjusted mean in at least one total domain score of the domains attention, executive function, verbal memory, or as a score of ≥3 in visuoconstruction (Shulman, 2000; Müller-Gerards et al., 2019). For details of domain construction see Müller-Gerards et al. (2019).

### Cognitive diagnoses

The MCI diagnosis was based on meeting all of the following published MCI criteria (Winblad et al., 2004): (1) cognitive impairment in at least one of the four domains reported above; (2) subjective cognitive decline (SCD) assessed with the question “In comparison to 2 years ago would you rate your memory function as better, same, or worse?” (SCD was defined as present if the participant’s answer was “worse”); (3) normal functional abilities and daily activities; (4) no dementia diagnosis. To examine incident MCI at t2 in cognitively healthy participants at t1, participants with dementia, MCI or with an objective cognitive impairment without fulfilling the obligate SCD criterion for MCI diagnosis were excluded as detailed above (see Figure 2). Participants at t2 not meeting MCI criteria as detailed above were categorized as “no MCI.”

Dementia diagnosis was defined as a physician’s previous diagnosis of dementia, meeting the DSM-IV (American Psychiatric Association, 1994) dementia diagnosis criteria or taking cholinesterase inhibitors [anatomic-therapeutic-chemical classification (ATC) issued by the World Health Organization, ATC code: N06DA] or other anti-dementia drugs (N06DX).

### Depressive symptoms

We assessed depressive symptoms using the German 15-item short form of the CES-D (Radloff, 1977; Hautzinger and Bailer, 1993). It was developed by The National Institute of Mental Health for epidemiological research and showed good sensitivity and specificity and high internal consistency across wide age ranges (Radloff and Teri, 1986; Lewinsohn et al., 1997) including in participants with cognitive impairment (Lewinsohn et al., 1997). The scale assesses the number of symptoms/week associated with depression, such as depressed mood, restless sleep, and feeling lonely. The answers are rated on a Likert scale with four possible categories: “0” = “rarely or none of the time (less than 1 day),” “1” = “sometimes (1–2 days),” “2” = often (3–4 days), and “4” = “almost or all of the time (5–7 days).” Scores range from 0 to 45 in the total t1 sample with higher scores indicating more depressive symptoms. In our final sample, scores range from 0 to 39. For up to three missing answers, missing values were replaced by the mean value of this item. The CES-D is considered an indicator of depression and is highly correlated with a clinical diagnosis of depression (Lewinsohn et al., 1997). A cut-off value of ≥17 was chosen to indicate clinically relevant depressive symptoms (Beekman et al., 1997; Icks et al., 2013; Engel et al., 2020). As mentioned above, the CES-D was assessed at t0, t1, and t2 in the study center. Furthermore, participants received three CES-D postal questionnaires between t1 and t2 and additional three CES-D postal questionnaires after t2 to assess the development of depressive symptoms over time (see Figure 1). Thus, we are able to analyze the course of depressive symptoms over a period of 13 years [for details see Engel et al. (2020)].

Besides using the dichotomized score to define depressive symptoms, participants were grouped into subgroups depending on their intra-individual mean value of the CES-D score form all available time-points (“overall CES-D mean”; minimum two CES-D measurements available) (Engel et al., 2020). The corresponding SD was calculated to show the variation of the CES-D score over time for each participant. Based on these two values (individual mean and SD), the population was stratified into four subgroups: stable low (individual SD < 6.8; overall CES-D mean < 13.6), stable high (individual SD < 6.8; overall CES-D mean > 20.4), stable around cut-off (≥17) for elevated depressive symptoms (individual SD < 6.8; CES-D mean ≥ 13.6; ≤ 20.4) and large fluctuations (individual SD ≥ 6.8; CES-D mean not used for definition of this group). For details see Engel et al. (2020).

### Covariates

Computer-assisted interviews at both time points collected information about medical/family history, coronary heart disease, smoking, and socio-economic status (Schmermund et al., 2002; Stang et al., 2005). The continuous education variable was stratified into four categories, with the highest category of ≥18 years of education and the lowest category of ≤10 years. Blood pressure was measured seated with an automated oscillometric blood pressure device (Omron, HEM-705CP, Mannheim, Germany), using the mean of the second and third value of three measurements (Schmermund et al., 2002) and classified according to JNC-7 [Joint National Committee on Prevention, Detection, Evaluation, and Treatment of High Blood Pressure; systolic blood pressure and/or diastolic blood pressure: normal (<120 mmHg and <80 mmHg), pre-hypertension (120–139 mmHg or 80–89 mmHg), stage 1 (140–159 mmHg or 90–99 mmHg) and stage 2 (≥160 mmHg or ≥100 mmHg or intake of antihypertensive medication)] (Chobanian et al., 2003). Standardized measured height and weight were used for calculating body mass index (BMI; kg/m^2^). Diabetes mellitus was defined present if participants reported a diagnosis of diabetes, used anti-diabetic medication or had a fasting glucose of >126 mg/dl (Icks et al., 2008). All participants were asked about known coronary heart disease and stroke using a physician-based questionnaire. “Current smoking” was defined as a history of cigarette smoking during the past year. If there was a history of smoking (longer than 1 year ago), participants were defined as “former smoker,” if there was no history of smoking as “never smoked.” Participants were asked to bring their medication in the original packing or the package insert to the appointment. Medication use was recorded according to ATC classification codes, including use of antidepressants (ATC code N06A). Cardio-Metabochip BeadArrays were used for genotyping of two single nucleotide polymorphisms (SNPs, rs7412 and rs429358) to discriminate between the *APOE* alleles ε2, ε3, and ε4. Participants defined as *APOE* ε4 positive had at least one allele 4 (ε2/ε4, ε3/ε4, and ε4/ε4). All other participants were defined as *APOE* ε4 negative.

The variables used for model adjustment were selected using a directed acyclic graph (DAG) based on current literature on the topic. The DAG was constructed using the online software DAGitty v3.0 (Textor et al., 2016). The minimally sufficient adjustment set included the following variables: age, sex, education, diabetes mellitus, coronary heart disease, and stroke (see Supplementary Figure 1).

### Statistical analyses

To compare participants with incident MCI vs. no MCI at t2 regarding sociodemographic and clinical characteristics, we performed Mann–Whitney *U* tests for continuous variables and Pearson χ^2^ tests for categorical variables. We used a log-linear regression model with a Poisson working likelihood to obtain relative risks (RR) and corresponding 95% confidence intervals (CI). The outcome variable was incident MCI at t2, exposure variables were depressive symptoms (CES-D score at t1) and CES-D subgroups over time (“stable low” as reference category). Because only 27 (1%) participants (5 incident MCI vs. 22 no MCI) showed stable high CES-D values over time, we decided to use three CES-D subgroups stable low [*n* = 2,157 (87%)], stable high or around cut-off [*n* = 149 (6%)], large fluctuations [*n* = 173 (7%)] for our analyses.

For each exposure of interest, we calculated three models: first, a crude model with only the exposure as an independent variable; second, a model additionally adjusted for age, sex, and education; third, a fully adjusted model including all variables suggested by the DAG.

## Results

Demographic and clinical characteristics of all study participants and stratified by MCI status at t2 are shown in Table 1. Participants with incident MCI at t2 were significantly older, had lower education levels, were more often *APOE* ε4 carrier and had more often a positive history of stroke than participants without incident MCI. Regarding depressive symptoms, participants with incident MCI at t2 showed higher CES-D scores at t1 and at t2, had more often CES-D scores above the cut-off at t1 and t2, showed a higher intake of antidepressants at t1 and had more often stable high or stable around cut-off CES-D scores since t0 than participants with no MCI at t2.

**TABLE 1:** Demographic and clinical characteristics of all study participants and stratified by incident mild cognitive impairment (MCI) vs. no MCI at t2.

	Total (*n* = 1547)	Incident MCI at t2 (*n* = 147)	No MCI at t2 (*n* = 1400)	*P-value* ^1^
Age, t1, years	62.7 ± 6.1	65.6 ± 7.6	62.4 ± 7.0	**<0.001**
Sex				
Male	724 (47)	71 (48)	653 (47)	0.70
Education				
≤10 years	107 (7)	14 (10)	93 (7)	
11–13 years	824 (53)	74 (50)	750 (54)	**0.013**
14–17 years	358 (23)	45 (31)	313 (22)	
≥18 years	258 (17)	14 (9)	244 (17)	
APOE ε4^2^				
ε4 positive	377 (26)	47 (33)	330 (25)	**0.026**
BMI, t1, kg/m^2^	27.8 ± 4.6	27.9 ± 5.0	27.8 ± 4.6	0.97
Prevalence of diabetes mellitus, t1	226 (15)	25 (17)	201 (14)	0.39
Blood pressure, t1				
Systolic blood pressure, mmHg	131.0 ± 18.7	131.9 ± 18.3	130.9 ± 18.8	0.46
Diastolic blood pressure, mmHg	78.9 ± 10.1	77.3 ± 8.9	79.0 ± 10.2	0.10
Prevalence of hypertension, t1				
JNC 7 stage 1^3^	390 (25)	36 (25)	354 (25)	0.98
JNC 7 stage 2^4^	118 (8)	11 (8)	107 (8)	
History of coronary heart disease, t1	91 (6)	12 (8)	79 (6)	0.22
History of stroke, t1	38 (3)	8 (5)	30 (2)	**0.014**
Smoking status, t1				
Never smoked	652 (42)	67 (46)	585 (42)	0.67
Former smoker	653 (42)	59 (40)	594 (42)	
Current smoker	242 (16)	21 (14)	221 (16)	
Depression				
CES-D^5^ score, t1	7.0 ± 6.0	9.4 ± 7.1	6.8 ± 5.8	**<0.001**
CES-D^6^ score, t2	6.9 ± 6.1	9.8 ± 7.6	6.6 ± 5.8	**<0.001**
CES-D^5^ ≥ 17, t1	111 (7)	20 (14)	91 (7)	**0.001**
CES-D^6^ ≥ 17, t2	111 (7)	21 (15)	90 (7)	**<0.001**
Use of antidepressants, t1	92 (6)	15 (10)	77 (6)	**0.022**
Use of antidepressants, t2	115 (7)	13 (9)	102 (7)	0.49
CES-D fluctuations over 13 years				
Stable low	1339 (86)	113 (77)	1226 (88)	**0.002**
Stable high	16 (1)	3 (2)	13 (1)	
Stable around cut-off	75 (5)	15 (10)	60 (4)	
Large fluctuations	117 (8)	16 (11)	101 (7)	

APOE, Apolipoprotein E; BMI, body mass index (kg/m^2^); JNC 7, Joint National Committee on Prevention, Detection, Evaluation, and Treatment of High Blood Pressure; MCI, Mild cognitive impairment; t1, first follow-up; t2, second follow-up.

Data are presented as means (±SD) or as numbers (%) unless otherwise indicated.

^1^Comparisons between groups (incident MCI at t2 vs. no MCI at t2) calculated using Mann–Whitney *U* test or Pearson χ^2^-test as appropriate; significant *p*-values are presented in bold.

^2^ε4 positive = at least one ε4 allele (ε2/ε4, ε3/ε4, and ε4/ε4); genotyping not available for *n* = 72 participants.

^3^Stage 1 = 140–159 mmHg.

^4^Stage 2 ≥ 160 mmHg.

^5^Missing values for *n* = 9 participants.

^6^Missing values for *n* = 45 participants.

Regression analyses revealed a significantly elevated RR for incident MCI per CES-D point increase for the total cohort (RR 1.053, 95% CI 1.031–1.076, all reported RR and 95% CI in the results section are fully adjusted unless otherwise indicated) and in men (1.046, 1.012–1.081) and women (1.059, 1.029–1.090) separately (see Table 2, upper part). When applying the CES-D cut-off of ≥17 points indicating clinically relevant depressive symptoms (see Table 2, lower part), we found a 2.22 (1.38–3.58) fold increased risk for incident MCI in the total sample. When stratifying by sex, we found a 2.59 (1.46–4.58) fold increased risk for incident MCI in women, but no increased MCI risk for men (1.29, 0.52–3.21, unadjusted). Looking at CES-D fluctuation patterns over 13 years (Table 3), stable high or stable around cut-off CES-D values showed a 2.27 (1.38–3.75) fold increased risk for incident MCI in the total cohort. Large fluctuations of CES-D scores were also associated with a 1.89 (1.10–3.23) fold increased MCI risk. When stratifying by sex, we found a 2.93 (1.59–5.41) increased risk for incident MCI in women with stable high or stable around cut-off CES-D scores and a 1.78 (0.89–3.55) fold increased risk for large fluctuations. Due to only five incident MCI cases in men, we cannot adjust our models and found a crude RR of 1.68 (0.68–4.19) for incident MCI in men with stable high or stable around cut-off CES-D scores.

**TABLE 2:** Association between Center for Epidemiologic Studies Depression Scale (CES-D) score at t1 and CES-D ≥ 17 at t1 and incident mild cognitive impairment (MCI) at t2 for the total sample and stratified by sex.

Relative risk for incident MCI at t2								

No MCI at t2	Incident MCI at t2	CES-D^1^ score at t1	Unadjusted RR (95% CI)	*P-value*	Adjusted (1) RR (95% CI)	*P-value*	Adjusted (2) RR (95% CI)	*P-value*
Total^1^								
*n* = 1392	*n* = 146	Increase by one point	1.052 (1.030–1.074)	**<0.001**	1.054 (1.032–1.077)	**<0.001**	1.053 (1.031–1.076)	**<0.001**
Men								
*n* = 651	*n* = 71	Increase by one point	1.044 (1.010–1.078)	**0.010**	1.046 (1.013–1.081)	**0.006**	1.046 (1.012–1.081)	**0.007**
Women								
*n* = 741	*n* = 75	Increase by one point	1.060 (1.031–1.089)	**<0.001**	1.061 (1.031–1.091)	**<0.001**	1.059 (1.029–1.090)	**<0.001**

		**CES-D^1^ ≥ 17 at t1**						

Total^1^								
*n* = 1301	*n* = 126	No	1.00 (reference)		1.00 (reference)		1.00 (reference)	
*n* = 91	*n* = 20	Yes	2.04 (1.27–3.27)	**0.003**	2.24 (1.39–3.61)	**0.001**	2.22 (1.38–3.58)	**0.001**
Men								
*n* = 616	*n* = 66	No	1.00 (reference)		1.00 (reference)		1.00 (reference)	
*n* = 35	*n* = 5	Yes	1.29 (0.52–3.21)	0.58	N/A^2^		N/A^2^	
Women								
*n* = 685	*n* = 60	No	1.00 (reference)		1.00 (reference)		1.00 (reference)	
*n* = 56	*n* = 15	Yes	2.62 (1.49–4.62)	**0.001**	2.68 (1.52–4.73)	**0.001**	2.59 (1.46–4.58)	**0.001**

MCI, mild cognitive impairment; CES-D, Center for Epidemiologic Studies Depression Scale; RR, relative risk; CI, confidence interval, N/A, not applicable.

Data are from log-linear regression models with a Poisson working likelihood; significant *p*-values are presented in bold.

(1) Adjusted for age at t1, sex (only for the total sample calculations: male/female) and years of education (≤10 years, 11–13 years, 14–17 years, ≥18 years).

(2) Additionally adjusted for diabetes mellitus at t1 (yes/no), history of coronary heart disease at t1 (yes/no) and history of stroke at t1 (yes/no).

^1^Missing values for *n* = 9 participants.

^2^No calculation due to small sample sizes of incident MCI cases.

**TABLE 3:** Association between Center for Epidemiologic Studies Depression Scale (CES-D) fluctuations over 13 years and incident mild cognitive impairment (MCI) at t2 for the total sample and stratified by sex.

Relative risk for incident MCI at t2								

No MCI at t2	Incident MCI at t2	CES-D fluctuations over 13 years	Unadjusted RR (95% CI)	*P-value*	Adjusted (1) RR (95% CI)	*P-value*	Adjusted (2) RR (95% CI)	*P-value*
Total								
*n* = 1226	*n* = 113	Stable low	1.00 (reference)		1.00 (reference)		1.00 (reference)	
*n* = 101	*n* = 16	Large fluctuations	1.62 (0.96–2.74)	**0.07**	1.95 (1.14–3.31)	**0.014**	1.89 (1.10–3.23)	**0.021**
*n* = 73	*n* = 18	Stable high or stable around cut-off	2.34 (1.43–3.85)	**0.001**	2.29 (1.39–3.77)	**0.001**	2.27 (1.38–3.75)	**0.001**
Men								
*n* = 596	*n* = 61	Stable low	1.00 (reference)		1.00 (reference)		1.00 (reference)	
*n* = 30	*n* = 5	Large fluctuations	1.54 (0.62–3.83)	0.35	N/A^1^		N/A^1^	
*n* = 27	*n* = 5	Stable high or stable around cut-off	1.68 (0.68–4.19)	0.26				
Women								
*n* = 630	*n* = 52	Stable low	1.00 (reference)		1.00 (reference)		1.00 (reference)	
*n* = 71	*n* = 11	Large fluctuations	1.76 (0.92–3.37)	0.09	1.96 (1.01–3.82)	**0.046**	1.78 (0.89–3.55)	0.10
*n* = 46	*n* = 13	Stable high or stable around cut-off	2.89 (1.57–5.31)	**0.001**	2.89 (1.57–5.32)	**0.001**	2.93 (1.59–5.41)	**0.001**

MCI, mild cognitive impairment; CES-D, Center for Epidemiologic Studies Depression Scale; RR, relative risk; CI, confidence interval, N/A, not applicable.

Data are from log-linear regression models with a Poisson working likelihood; significant *p*-values are presented in bold.

(1) Adjusted for age at t1, sex (only for the total sample calculations: male/female) and years of education (≤10 years, 11–13 years, 14–17 years,≥18 years).

(2) Additionally adjusted for diabetes mellitus at t1 (yes/no), history of coronary heart disease at t1 (yes/no) and history of stroke at t1 (yes/no).

^1^No calculation due to small sample sizes of incident MCI cases.

## Discussion

In our population-based cohort, depressive symptoms on the CES-D in cognitively unimpaired participants were associated with an increased risk of incident MCI 5 years later in both sexes. When applying the cut-off value for clinically relevant depressive symptoms, we found a 2.6-fold increased MCI risk for women. The same pattern was seen regarding fluctuations of depressive symptoms over 13 years: We found stable high or stable around cut-off scores on the CES-D to be associated with a nearly threefold increased risk of incident MCI in women. In addition, large fluctuations of depressive symptoms over time were associated with a nearly two-fold increased risk of incident MCI.

There is strong evidence on the association between depression and incident dementia showing an approximately twofold increased risk (Diniz et al., 2013; Cherbuin et al., 2015; Ford et al., 2018; Livingston et al., 2020). Depression is a contributor to increased dementia risk accounting for 4% of dementia cases worldwide, which might be prevented or delayed by elimination of this risk factor (Livingston et al., 2020). Our results regarding depressive symptoms and incident MCI in cognitively normal individuals are in line with the majority of studies (Barnes et al., 2006; Geda et al., 2006; Goveas et al., 2011; Steenland et al., 2012; Lara et al., 2017). Depressed individuals show a hyperactivity of the central stress response system that increases production of the stress hormones glucocorticoids (Holsboer, 2000). High levels of glucocorticoids lead to damage of the hippocampus (Conrad, 2008) and higher glucocorticoid levels have been associated with impaired cognitive functioning in both healthy and depressed individuals (Gomez et al., 2006), especially in memory and executive functioning (Rogers et al., 2004). The importance of the HPA-axis homeostasis for cognition is further supported by evidence of hypocortisolemia in depression that may occur after a prolonged period of hyperactivity of the HPA-axis (Fries et al., 2005; Miller et al., 2007). Low levels of corticosteroids might have a negative effect on cognition as well (Belanoff et al., 2001). Further biological mechanisms linking depression to dementia include inflammatory changes (Dowlati et al., 2010) and increased amyloid production and deposition of amyloid plaques (Jack and Holtzman, 2013). The association between depressive symptoms and the development of dementia has led to an ongoing debate on the direction of causality in this association (Dafsari and Jessen, 2020). Depression might be a risk factor for dementia, an early sign or prodromal stage of dementia, or occurring in the state of cognitive impairment/dementia as a reaction to cognitive or functional disabilities. Because our depression scores were assessed when participants were cognitively unimpaired, it can be assumed that our data indicate toward depressive symptoms as a risk factor for MCI and dementia in the further course. We cannot strictly rule out that depressive symptoms might also be a prodromal symptom of MCI in some of our participants. Regardless, both scenarios have strong implications. Depression is common among elderly individuals. Thus, identification of depression at an early time point and adequate treatment according to the depression guideline (Deutsche Gesellschaft für Psychiatrie und Psychotherapie, Psychosomatik und Nervenheilkunde) (DGPPN et al., 2015) might delay cognitive decline in the future.

Because cerebrovascular disease may lead to structural damage in frontal-striatal or frontal-executive circuits (Rashidi-Ranjbar et al., 2020) which is associated with depressive symptoms, we controlled our models for vascular conditions. This did not change our results.

Regarding symptom fluctuation over 13 years, we found stable high or stable around cut-off CES-D values, but also large fluctuations over time to be associated with a twofold increased risk of MCI. Only few studies examined depressive symptom fluctuations and the development of MCI. Dotson et al. (2010) found that recurrent elevated depressive symptoms increase the risk for all-cause dementia and AD as a function of the number of episodes. However, recurrence of depressive symptoms did not increase risk of incident MCI in their cohort. In our cohort, the RR of stable high or stable around cut-off CES-D scores over time and MCI were higher than the risks for large fluctuations or clinically relevant CES-D scores at one time point. This finding is in line with the postulated pathophysiological mechanisms linking depression and cognitive decline since repeated high depressive scores over time would result in repeated or continuous insult, for example to the hippocampus (Sapolsky et al., 1986). However, single episodes of depressive symptoms (as indicated by large fluctuations or by the CES-D score at t1) might also enhance the vulnerability to the adverse impact of depressive symptoms on cognitive functioning and brain integrity (Geda et al., 2006; Barnes et al., 2012; Dlugaj et al., 2015). This is also in line with a recent study showing early adulthood depressive symptoms to be a risk factor for cognitive impairment independent of mid- or late-life depressive symptoms (Brenowitz et al., 2021).

The stratification of our MCI group in amnestic (aMCI) and non-amnestic MCI (naMCI) reflecting different underlying dementia etiologies [aMCI more likely to progress to AD, naMCI more likely to progress to vascular dementia or other non-AD dementia (Winblad et al., 2004)] showed a similar association with both subtypes (see Supplementary Tables 1, 2). The association with aMCI was more pronounced. The association with both subtypes of MCI seems plausible. For example, it has been shown that glucocorticoids promote amyloid-beta formation in the mouse model of AD (Green et al., 2006). Regarding naMCI, studies showed higher depressive symptoms in participants with cardiovascular diseases (Byers and Yaffe, 2011; Taylor et al., 2013). The same pattern was also found in our previous cross-sectional analysis of MCI subtypes and depression (Dlugaj et al., 2015).

Despite the sex-independent association of depressive symptoms with MCI 5 years later, the association of clinically relevant depressive symptoms was evident only in women. Thus, depressive symptoms seem to increase the risk of MCI in both sexes without the necessity of reaching the level of clinically relevant depressive symptoms. It is known that the prevalence of depression is higher in women (Leach et al., 2008). In our cohort, only 40 (out of 722) men showed clinically relevant depressive symptoms. Only five men with clinically relevant depressive symptoms developed MCI over time. Thus, our models might not have been able to detect associations between clinically relevant depressive symptoms and MCI in men. The same is true for CES-D fluctuations over 13 years with only 32 men with stable high or stable around cut-off CES-D values and 10 MCI cases over time. Although women seem to have a higher lifetime prevalence of depression (Bekker and van Mens-Verhulst, 2007), sex differences seem to persist mainly before menopause (Cyranowski et al., 2000; Cairney and Wade, 2002). However, not all reported sex differences seem to result from circulation gonadal hormones (Bangasser and Cuarenta, 2021). More research is needed to fully understand the circuits and mechanisms that contribute to sex differences in depression as this has implications for adequate treatments for both men and women.

Our study has several strengths. We have examined a large, well-characterized, population-based sample that enabled us to include only cognitively unimpaired participants at a specific time point and examine incident MCI cases 5 years later. The cognitive assessment at t1 that has shown a good accuracy in identifying participants with MCI in a previous study (Wege et al., 2011), and therefore allows a good identification of incident MCI cases. The availability of multiple measurements of depressive symptoms makes it possible to investigate patterns of depressive symptoms over 13 years. There are some limitations that need to be considered. Because this is a longitudinal study with cognitive data assessed at the first and second follow-up examination, cognitively impaired participants might have been less likely to participate resulting in a cohort representing a healthier population. Depression was not diagnosed by a clinician. Thus, we do not have a clinical depression diagnosis or knowledge about the beginning of symptoms. However, the CES-D is a widely utilized and well-established instrument to measure depressive symptoms. Furthermore, we used a well-established cut-off to define clinically relevant depressive symptoms in our cohort (Lewinsohn et al., 1997). Regarding fluctuations of depressive symptoms, not all participants had the same number of CES-D measurements over time. Finally, we do not have any biomarker information to differentiate between underlying pathologies for incident MCI and had small sample sizes of men with incident MCI and depressive symptoms.

In summary, an increase in depressive symptoms in cognitively unimpaired participants was associated with an increased risk of incident MCI after 5 years. Our findings contribute to the knowledge about depression and cognitive decline indicating depression to be a risk factor for MCI and for dementia in the further course. Assessing depressive symptoms and taking them seriously when reported might help to identify individuals at risk of cognitive decline in order to enable early treatment to delay dementia onset.

## Data availability statement

The raw data supporting the conclusions of this article will be made available by the authors, upon resonable request.

## Ethics statement

The studies involving human participants were reviewed and approved by the Ethics Committee of the Faculty of Medicine, University of Duisburg-Essen, Essen, Germany. The patients/participants provided their written informed consent to participate in this study.

## Author contributions

All authors listed have made a substantial, direct, and intellectual contribution to the work, and approved it for publication.
