# Supplementary material for: Fluctuation of depressive symptoms in cognitively unimpaired participants and the risk of mild cognitive impairment 5 years later: Results of the Heinz Nixdorf Recall study
Source: Front Behav Neurosci. 2022 Oct 25;16:988621. doi: 10.3389/fnbeh.2022.988621 (PMC9640513; doi:10.3389/fnbeh.2022.988621)
Supplement: Supplementary file 1 [file Data_Sheet_1.docx]

**Supplementary material:**

**Fluctuation of depressive symptoms in cognitively unimpaired participants and the risk of MCI five years later: Results of the Heinz Nixdorf Recall study**

Martha Jokisch^1*^, Sara Schramm^2^, Christian Weimar^2,3^, Susanne Moebus^4^, Janine Gronewold^1^, Nico Dragano^5^, Karl-Heinz Jöckel^2^ for the Heinz Nixdorf Recall Study Investigative Group.

1. Department of Neurology, University Hospital of Essen, University of Duisburg-Essen, Essen, Germany.
2. Institute for Medical Informatics, Biometry, and Epidemiology, University Hospital Essen, University of Duisburg-Essen, Essen, Germany.
3. BDH-Klinik Elzach gGmbH, Elzach, Germany.
4. Institute for Urban Public Health, University Hospital Essen, University of Duisburg-Essen, Essen, Germany.
5. Institute of Medical Sociology, Centre for Health and Society, Medical Faculty, University of Düsseldorf, Düsseldorf, Germany.

***Corresponding author:**

Martha Jokisch, PhD

University Hospital Essen, Department of Neurology

Hufelandstr. 55, 45147 Essen, Germany

Phone number: +49 201 723 2588

Fax number: +49 201 723

E-mail: Martha.Jokisch@uk-essen.de


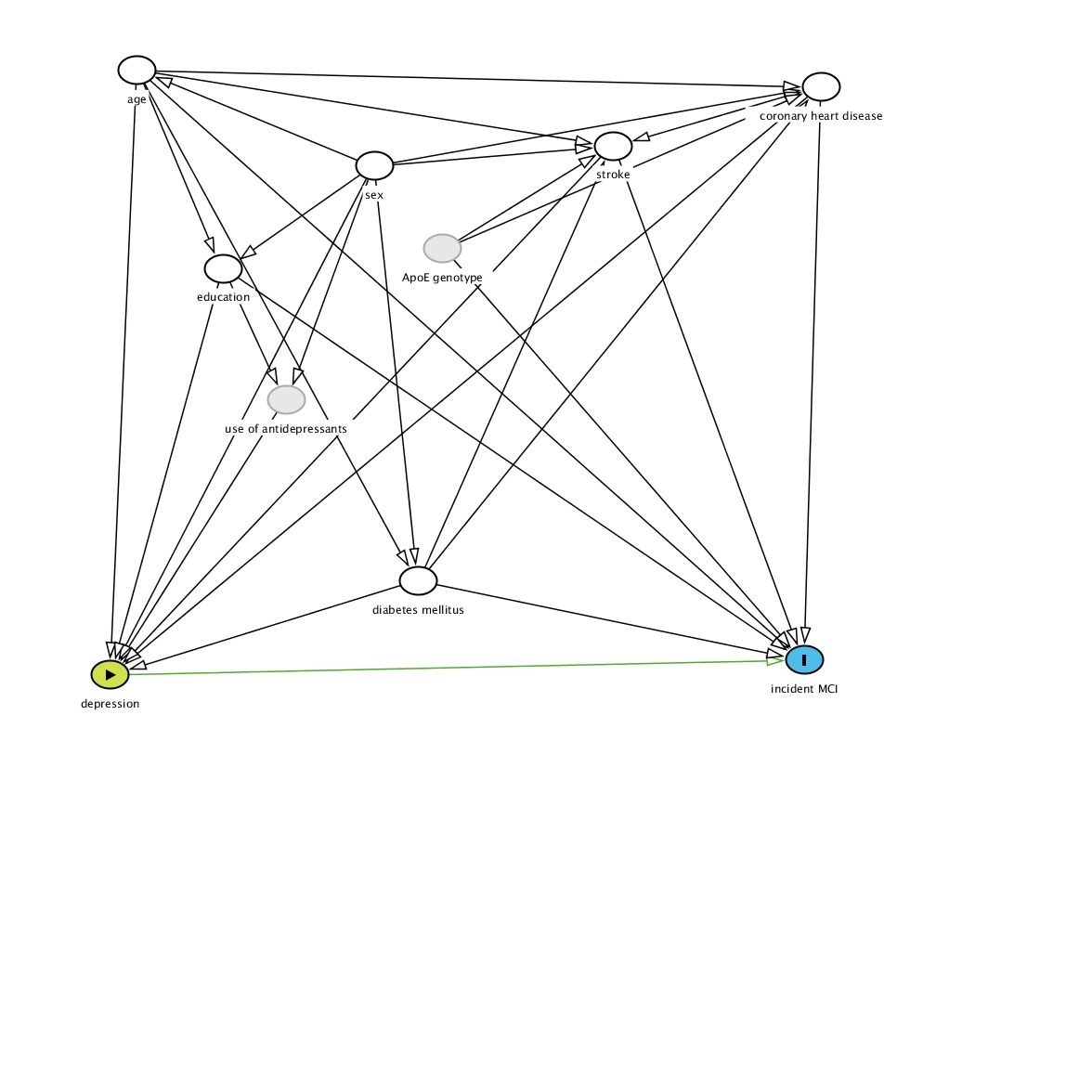


**Supplemental Figure 1.** Directed acyclic graphs (DAGs) used to determine the confounding covariates for the regression analyses adjustment sets of this study. The exposure of interest is marked in green; the outcome of interest is marked in blue; confounders are marked in white; all other variables are marked in gray. Interactions between variables are indicated by arrows. Source: Created with DAGitty v3.0

**Supplemental Table 1.** Association between CES-D score at t1, CES-D ≥ 17at t1, and CES-D fluctuations over 13 years and incident amnestic MCI at t2 for the total sample

| **Relative risk for incident amnestic MCI at t2** | | |  |  |  |  |  |  |
| --- | --- | --- | --- | --- | --- | --- | --- | --- |
| No MCI at t2 | Incident aMCI at t2 | **CES-D^1^ score at t1** | Unadjusted |  | Adjusted (1) |  | Adjusted (2) |  |
|  |  |  | RR (95% CI) | p value | RR (95% CI) | p value | RR (95% CI) | p value |
|  |  |  |  |  |  |  |  |  |
| n=1392^1^ | n=49 | **Increase by one point** | 1.075 (1.04-1.11) | **<0.001** | 1.08 (1.048-1.12) | **<0.001** | 1.08 (1.047-1.12) | **<0.001** |
|  |  |  |  |  |  |  |  |  |
|  |  | **CES-D^1^ ≥ 17** |  |  |  |  |  |  |
| n=1301^1^ | n=42 | No | 1.00 (reference) |  | 1.00 (reference) |  | 1.00 (reference) |  |
| n=91 | n=7 | Yes | 2.28 (1.03-5.08) | **0.043** | 2.63 (1.17-5.92) | **0.020** | 2.63 (1.16-5.94) | **0.020** |
|  |  |  |  |  |  |  |  |  |
|  |  | **CES-D fluctuations over 13 years** |  |  |  |  |  |  |
|  |  |  |  |  |  |  |  |  |
|  |  |  |  |  |  |  |  |  |
| n=1226 | n=35 | Stable low | 1.00 (reference) |  | 1.00 (reference) |  | 1.00 (reference) |  |
| n=101 | n=7 | Large fluctuations | 2.34 (1.04-5.26) | **0.041** | 2.93 (1.27-6.76) | **0.012** | 2.75 (1.18-6.39) | **0.019** |
| n=73 | n=7 | Stable high or stable around cut-off | 3.15 (1.40-7.10) | **0.006** | 2.98 (1.31-6.75) | **0.009** | 2.99 (1.32-6.81) | **0.009** |

Abbreviations: aMCI = amnestic mild cognitive impairment; CES-D = Center for Epidemiologic Studies Depression Scale; RR =relative risk; CI = confidence interval

Data are from log-linear regression models with a Poisson working likelihood; significant p values are presented in bold

(1) Adjusted for age at t1, gender (only for the total sample calculations: male/female) and years of education (≤10 years, 11-13 years, 14-17 years, ≥18 years)

(2) Additionally adjusted for diabetes mellitus at t1 (yes/no), history of coronary heart disease at t1 (yes/no) and history of stroke at t1 (yes/no)

^1^Missing values for n=8 no MCI at t2 participants

**Supplemental Table 2.** Association between CES-D score at t1, CES-D ≥ 17at t1, and CES-D fluctuations over 13 years and incident non-amnestic MCI at t2 for the total sample

| **Relative risk for incident non-amnestic MCI at t2** | | |  |  |  |  |  |  |
| --- | --- | --- | --- | --- | --- | --- | --- | --- |
| No MCI at t2 | Incident naMCI at t2 | **CES-D^1^ score at t1** | Unadjusted |  | Adjusted (1) |  | Adjusted (2) |  |
|  |  |  | RR (95% CI) | p value | RR (95% CI) | p value | RR (95% CI) | p value |
|  |  |  |  |  |  |  |  |  |
| n=1392^1^ | n=97^1^ | **Increase by one point** | 1.04 (1.015-1.072) | **0.002** | 1.04 (1.014-1.072) | **0.004** | 1.04 (1.013-1.072) | **0.004** |
|  |  |  |  |  |  |  |  |  |
|  |  | **CES-D^1^ ≥ 17** |  |  |  |  |  |  |
| n=1301 | n=84 | No | 1.00 (reference) |  | 1.00 (reference) |  | 1.00 (reference) |  |
| n=91 | n=13 | Yes | 2.06 (1.15-3.70) | **0.015** | 2.19 (1.22-3.95) | **0.020** | 2.16 (1.20-3.90) | **0.010** |
|  |  |  |  |  |  |  |  |  |
|  |  | **CES-D fluctuations over 13 years** |  |  |  |  |  |  |
|  |  |  |  |  |  |  |  |  |
|  |  |  |  |  |  |  |  |  |
| n=1226 | n=78 | Stable low | 1.00 (reference) |  | 1.00 (reference) |  | 1.00 (reference) |  |
| n=101 | n=9 | Large fluctuations | 1.37 (0.69-2.73) | 0.37 | 1.67 (0.83-3.35) | 0.15 | 1.65 (0.82-3.34) | 0.16 |
| n=73 | n=11 | Stable high or stable around cut-off | 2.19 (1.17-4.12) | **0.015** | 2.09 (1.11-3.94) | **0.023** | 2.06 (1.09-3.88) | **0.026** |

Abbreviations: naMCI = non-amnestic mild cognitive impairment; CES-D = Center for Epidemiologic Studies Depression Scale; RR =relative risk; CI = confidence interval

Data are from log-linear regression models with a Poisson working likelihood; significant p values are presented in bold

(1) Adjusted for age at t1, gender (only for the total sample calculations: male/female) and years of education (≤10 years, 11-13 years, 14-17 years, ≥18 years)

(2) Additionally adjusted for diabetes mellitus at t1 (yes/no), history of coronary heart disease at t1 (yes/no) and history of stroke at t1 (yes/no)

^1^Missing values for n=9 participants (n=8 no MCI at t2 and n=1 naMCI at t2)
